# Are there any changes in burden and management of communicable diseases in areas affected by Cyclone Nargis?

**DOI:** 10.1186/1752-1505-5-9

**Published:** 2011-06-28

**Authors:** Nyan Win Myint, Jaranit Kaewkungwal, Pratap Singhasivanon, Kamron Chaisiri, Pornpet Panjapiyakul, Pichit Siriwan, Arun K Mallik, Soe Lwin Nyein, Thet Thet Mu

**Affiliations:** 1 Medical Care Division, Department of Health, Ministry of Health, Nay Pyi Taw, Myanmar; 2 Faculty of Tropical Medicine, Mahidol University, Bangkok, Thailand; 3 Permanent Secretary Office, Ministry of Public Health, Nonthaburi, Thailand; 4 Relief and Community Health Bureau, Red Cross Society, Bangkok, Thailand; 5 Inter-agency Coordination and Emergency and Humanitrian Action, WHO Office, Nonthaburi, Thailand; 6 Central Epidemiological Unit, Department of Health, Ministry of Health, Nay Pyi Taw, Myanmar; 7 Health Information Division, Department of Health Planning, Ministry of Health, Nay pyi Taw, Myanmar

## Abstract

**Background:**

This study aims to assess the situation of communicable diseases under national surveillance in the Cyclone Nargis-affected areas in Myanmar (Burma) before and after the incident.

**Methods:**

Monthly data during 2007, 2008 and 2009 from the routine reporting system for disease surveillance of the Myanmar Ministry of Health (MMOH) were reviewed and compared with weekly reporting from the Early Warning and Rapid Response (EWAR) system. Data from some UN agencies, NGOs and Tri-Partite Core Group (TCG) periodic reviews were also extracted for comparisons with indicators from Sphere and the Inter-Agency Standing Committee.

**Results:**

Compared to 2007 and 2009, large and atypical increases in diarrheal disease and especially dysentery cases occurred in 2008 following Cyclone Nargis. A seasonal increase in ARI reached levels higher than usual in the months of 2008 post-Nargis. The number of malaria cases post-Nargis also increased, but it was less clear if this reflected normal seasonal patterns or was specifically associated with the disaster event. There was no significant change in the occurrence of other communicable diseases in Nargis-affected areas. Except for a small decrease in mortality for diarrheal diseases and ARI in 2008 in Nargis-affected areas, population-based mortality rates for all other communicable diseases showed no significant change in 2008 in these areas, compared to 2007 and 2009. Tuberculosis control programs reached their targets of 70% case detection and 85% treatment success rates in 2007 and 2008. Vaccination coverage rates for DPT 3^rd ^dose and measles remained at high though measles coverage still did not reach the Sphere target of 95% even by 2009. Sanitary latrine coverage in the Nargis-affected area dropped sharply to 50% in the months of 2008 following the incident but then rose to 72% in 2009.

**Conclusion:**

While the incidence of diarrhea, dysentery and ARI increased post-Nargis in areas affected by the incident, the incidence rate for other diseases and mortality rates did not increase, and normal disease patterns resumed by 2009. This suggests that health services as well as prevention and control measures provided to the Nargis-affected population mitigated what could have been a far more severe health impact.

## Background

There are about 450 to 800 major natural disasters each year around the world; the impact of such disasters is exacerbated by a number of factors including global warming, increased population movement, environmental damage, poverty and inadequate or underfunded public health systems [1]. The typical effects of such disasters include injury, death, infectious diseases outbreaks, large-scale population displacement, disruption of essential services, destruction of property and infrastructure, economic loss and psychological harm [2,3]. The burden of excess morbidity and mortality caused by disasters may vary depending on the underlying characteristics of the disaster-affected population [4]. During disaster situations, communicable diseases can cause high mortality and morbidity due to disruption of health services, poor access to health care, malnutrition and inadequate supply of logistical necessities [5,6]. Disaster-affected people are particularly vulnerable to communicable diseases due to malnutrition, stress, fatigue and unsanitary living conditions [6].

Cyclone Nargis hit the delta area of Myanmar on 2 and 3 May, 2008, causing many deaths, destroying infrastructure, and affecting economic and social activities [7]. It was the most destructive natural disaster in recent history of Myanmar and the most deadly cyclone in Asia since 1991; 2.4 million people were severely affected by Nargis [7]. The most common water- and food-borne diseases in the affected area before Cyclone Nargis were diarrheal diseases including cholera, typhoid, hepatitis A and E and acute watery diarrhea and dysentery (shigellosis). Dengue and malaria had been the major vector-borne diseases endemic in the affected area. Measles, acute respiratory infection (ARI), diphtheria, pertussis and meningococcal diseases were reported as associated with the overcrowding in the area. Other diseases endemic in the region were tuberculosis, snake bites and sexually transmitted infections (STIs) [8].

The Myanmar Ministry of Health (MMOH) set up the Early Warning and Rapid Response (EWAR) surveillance system after Cyclone Nargis with involvement of other national and international agencies working in the Nargis-affected area and implemented it from the first week of June 2008 until May 2009 [9]. It included rumor verification for disease monitoring and management, particularly for early warning and rapid responses. It tracked 15 common diseases and conditions in the affected area: acute diarrhea, suspected cholera (acute watery diarrhea), bloody diarrhea, acute jaundice, ARI/pneumonia, suspected measles, suspected meningitis, malaria confirmed by rapid diagnostic test, suspected dengue, suspected dengue hemorrhagic fever, trauma cases, suspected tetanus, sexually transmitted infection, snake bites, and unexplained cluster of health events [9]. The United Nations, ASEAN and Myanmar government also set up a "Tripartite Core Group" (TCG) as the coordinating body to manage the response to Cyclone Nargis [10]. This study was carried out to examine communicable disease burden and the changes in morbidity and mortality of the diseases under national surveillance in the Cyclone Nargis-affected area. The study also attempted to assess the effectiveness of control measures for these diseases, employing health management information system indicators of the Sphere and Inter-Agency Standing Committee (IASC) for global health cluster communicable diseases [11,12].

## Materials and methods

### Study sites

Cyclone Nargis significantly affected 37 townships in Yangon and Ayeyarwaddy division [7]. The focus of data collection in this study included 10 of those 37 townships, all of which suffered the devastating effects of Cyclone Nargis. The ten townships included seven in Ayeyarwady division (Ngapudaw, Labutta, Bogale, Pyapon, Dedaye, Kyaiklat and Mawlamyinegyun townships) and three in Yangon division (Twantay, Kawnmu and Kungyangon townships). There were about 2.8 million people living in these ten townships in 2007 [13].

### Secondary data sources

To examine the communicable disease burden and changes in mortality and morbidity of diseases under national surveillance, system data were extracted for the specific study sites from the MMOH Health Management Information System (HMIS). The HMIS is the routine reporting system for 17 diseases under national surveillance in Myanmar, with monthly reporting from township to central level [13]. The official secondary data during 2007-2009 from the Department of Health, and Department of Health Planning regarding communicable diseases prevention and control programs such as immunization and water-sanitation program were also extracted for assessing the incidence of such communicable diseases at the township level. In addition, other related data such as distribution of relief items from Myanmar Red Cross Society and Myanmar offices (WHO, UNICEF, Save the Children, Merlin and MSF (Holland)) were also requested for exploring the strengths and weaknesses in communicable disease management. The 2008 data from the ten study townships are extracted and presented separately to compare the four months pre-Nargis to the eight months post-Nargis and for comparison with data from 2007 and 2009. Data from the periodic reviews released by Tri-partite Core Group (TCG) are also used for supplementation of population data [10].

### Primary data Sources

The study employed both quantitative and qualitative primary data collection methods. A questionnaire regarding public health emergency (PHE) preparedness, particularly relating to disease surveillance capacity, was developed and sent to hospitals under the Department of Health. The questionnaires were sent to 65 hospitals (25% of the total 252 hospitals in coastal Myanmar which include all 5 states/divisions (i.e., Yangon, Ayeyarwaddy, Mon, Tanintharyi and Rakhine). The study selected hospitals in coastal area of Myanmar because of the high risk for emergency and disaster in this area such as the tsunami in 2004, Cyclone Mala in 2006 and Cyclone Nargis in 2008. The questionnaires were developed based on questionnaires and guidelines from the World Health Organization (WHO), the Association of Professionals for Infection Control and Epidemiology (APIC) and the U.S. Centers for Disease Control and Prevention. Stratified random sampling methods based on referral level are used for the hospital survey on PHE preparedness because it is found that the preparedness level depends on the referral level [14]. Hospitals under Department of Health are classified as primary referral (station/sub-township hospitals), secondary referral (township/district hospitals) and tertiary (state/division/specialist hospitals) depending on the health services provided to the public. The questionnaires were completed by hospital directors from those hospitals by means of a self- administered method. Focus group discussion was used to learn more about community perceptions of the health sector response to Cyclone Nargis. A total of 6 focus group discussions were carried out in 3 villages severely affected by Cyclone Nargis: Amar and Kyan-ka-dune villages in Pyapone township and Mangalake village in Kungyankone township. In each village, two focus group discussions were conducted: one for community members and one for government personnel and community leaders. Each focus group included 6 to 9 persons, and the discussion time ranged from 65 to 95 minutes. Two facilitators and two note takers were used to carry out each focus group discussion. The detailed analyses of hospital preparedness and community perceptions will be reported elsewhere and only issues related to communicable diseases were presented in this paper.

### Ethical Consideration

This study received ethical approvals from Myanmar Ministry of Health and Faculty of Tropical Medicine, Mahidol University.

## Results

### Disease surveillance in study areas

MMOH set up the coordinating health sectors for disease surveillance, outbreak detection and response. Disease surveillance data for both the routine and the new EWAR systems were submitted from the local level to the centralized national health management information system (HMIS). The MMOH recognized that there were still challenges regarding timeliness of notification and complete data reporting in both HMIS and EWAR. Underreporting of cases is still a challenging issue for the regular reporting system because it relies on basic health staff for reporting of data; underutilization and difficulties in accessing health services in some rural areas are further reasons for under-reporting of diseases occurring in the population.

### Hospital-based surveillance

The routine HMIS surveillance system includes reporting from hospitals at all levels throughout the country. Table 1 shows the different kinds of surveillance reporting in 2009, one year after Cyclone Nargis, for primary, secondary and tertiary hospitals in cyclone-affected and unaffected areas of the country. As shown in the table, among the 40 out of 65 hospitals that responded to the survey, about 90% of the hospitals had a surveillance system for the 17 diseases under national surveillance and about 90% also had surveillance for abnormal diagnoses or deaths. More than 60% of the responding hospitals had a syndromic surveillance system, and only 32% had microbiological surveillance system, (with especially low percentage in primary referral hospitals, at less than 10%).

**Table 1:** Availability of different surveillance systems in Nargis and Non-Nargis areas in 2009

Variables	Hospitals in Nargis affected Area				Hospitals in Non-Nargis affected Area			
	
	Primary n = 12	Secondary n = 7	Tertiary n = 2	Total N = 21	Primary n = 11	Secondary n = 5	Tertiary n = 3	Total N = 19
	
	n (%)	n (%)	n (%)	n (%)	n (%)	n (%)	n (%)	n (%)
Diseases under national surveillance	10 (83%)	6 (86%)	2 (100%)	18(86%)	10 (91%)	4 (80%)	3(100%)	17(89%)

Microbiological surveillance	1 (8%)	3 (43%)	1(50%)	5 (24%)	1(9%)	3 (60%)	2 (67%)	6(32%)

Surveillance on abnormal diagnosis and death	9 (75%)	6 (86%)	1 (50%)	16 (76%)	10 (91%)	4 (80%)	3 (100%)	17 (89%)

Syndrome surveillance	5 (42%)	5 (71%)	2 (100%)	12(57%)	7 (6%)	4 (80%)	2 (67%)	13(68%)

### Diarrhea & Dysentery

**Figure 1:**
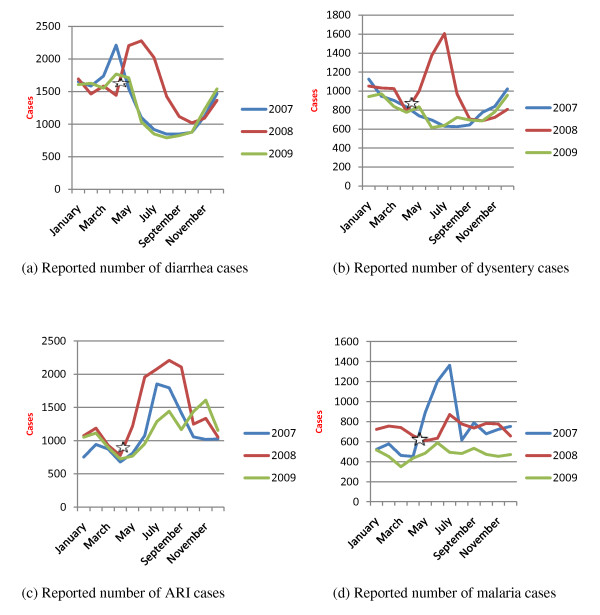
**Four leading communicable diseases before and after Cyclone Nargis in study townships**. (White Star): Cyclone Nargis

Among the communicable diseases reported in the HMIS, diarrhea, dysentery and acute respiratory infections (ARI) posed the highest post-Cyclone disease burden in the communities within the Nargis- affected areas, including the ten study townships (Figure 1, Table 2). The incidence rate for acute diarrhea before Cyclone Nargis was 571.4 per 100,000 persons per year in 2007 and 683.44 per 100,000 persons per year in the four pre-Nargis months of 2008; the rate increased to 798.81 in the post-Nargis months of 2008and declined in 2009 to 610.6 per 100,000 in the study area. As shown in the figure, the seasonal peak for diarrhea cases in 2007 and 2009, the years before and after Cyclone Nargis, were from March to May; in contrast, the 2008 peak for diarrhea cases was from May to July, immediately following the Nargis incident. EWAR Weekly Reports also showed that diarrhea cases were peaked during June and July 2008 after Cyclone Nargis. In contrast to the average of less than 5 reported cases per month of severe acute watery diarrhea (suspected cholera) cases per month, the number of reported suspected cholera cases was highest during June 2008 (21 cases) and surged again a year after Nargis in March 2009 (15 cases); according to EWAR reports following Cyclone Nargis. Increasing incidence of diarrhea coincided with deterioration in population sanitary latrine coverage in study townships in Nargis-affected areas (75.5% pre-Nargis vs 50.5% post-Nargis). The participants from focus group discussion also revealed that diarrhea diseases, ARI, influenza and pneumonia were prevalent immediately after Cyclone Nargis. One participant mentioned that

**Table 2:** Morbidity, mortality and case fatality rates of diseases under national surveillance in Nargis-affected townships, 2007 to 2009

Diseases	Morbidity(case rate per 100,000/year)				Mortality(case rate per 100,000/year)				Case fatality rate			
	
	2007	2008		2009	2007	2008		2009	2007	2008		2009
									
		Pre-Nargis	Post-Nargis			Pre-Nargis	Post-Nargis			Pre-Nargis	Post-Nargis	
Diarrhea	571.40	683.44	798.81	610.56	0.68	0.99	0.06	0.28	0.12	0.15	0.01	0.04

Dysentery	350.90	430.21	502.27	374.39	0.04	0.00	0.00	0.00	0.01	0.00	0.00	0.00

Food poisoning	6.57	9.06	5.36	13.27	0.18	0.11	0.06	0.08	2.73	1.22	1.19	0.60

Typhoid	7.83	4.53	3.76	3.17	0.04	0.11	0.00	0.00	0.46	2.44	0.00	0.00

Measles	5.47	0.00	0.00	0.00	0.00	0.00	0.00	0.00	0.00	0.00	0.00	0.00

Diphtheria	0.00	0.00	0.55	0.00	0.00	0.00	0.00	0.00	0.00	0.00	0.00	0.00

Whooping cough	0.00	0.00	0.00	0.00	0.00	0.00	0.00	0.00	0.00	0.00	0.00	0.00

Neonatal tetanus	0.00	0.00	0.00	0.00	0.00	0.00	0.00	0.00	0.00	0.00	0.00	0.00

ARI	4041.91	3711.64	7229.7	4661.59	4.86	3.74	2.76	4.11	0.12	0.10	0.04	0.09

Tetanus	0.47	0.55	0.64	0.79	0.11	0.00	0.06	0.00	23.08	0.00	10.00	0.00

Meningitis	0.50	0.33	0.11	0.36	0.04	0.11	0.06	0.00	7.14	33.33	33.33	0.00

Viral hepatitis	9.08	12.26	8.42	8.24	0.11	0.00	0.00	0.12	1.19	0.00	0.00	1.44

Rabies	0.29	0.44	0.06	0.24	0.29	0.44	0.06	0.24	100.00	100.00	100.00	100.00

Malaria	324.44	318.10	372.84	227.18	0.50	0.66	0.26	0.36	0.15	0.21	0.07	0.16

DHF	35.65	13.03	15.80	12.40	0.36	0.08	0.06	0.04	1.01	0.85	0.52	0.32

Snake bite	7.11	4.31	7.21	6.97	2.12	1.10	2.87	2.38	29.80	25.64	39.82	34.09

Sputum(+) tuberculosis	67.11	57.23	57.15	47.54	0.07	0.11	0.06	0.04	0.10	0.19	0.11	0.08

"Villagers suffered mostly diarrhea and common cold. Pneumonia was common especially among children. Dengue and malaria were not common among villagers." (35 years old woman)

Despite increasing disease incidence, mortality rates for diarrhea declined between 2007 (0.68 per 100,000 person year) and 2009 (0.06 in 2008 post- Nargis and 0.28 in 2009). This finding also corresponded to the high percentage of treatment with oral rehydration therapy (ORT) among children with diarrhea (>95%) in the health facilities and a decrease in severe dehydration among such children (2.31% in 2007 to <1% in 2008 and 2009) in study townships (Table 3). However, as per TCG a periodic report [10], ORT treatment among diarrhea patients in the community was around 50% after Cyclone Nargis. Overall, case fatality rate for diarrhea diseases declined from 0.12% in 2007 to 0.04% in 2009.

**Table 3:** Public health indicators of healthcare services and accessibility in 10 townships of Nargis affected area, 2007 to 2009

Indicators	2007 (Range)^ψ^	2008		2009 (Range)^ψ^
			
		Pre-Nargis (Range)^ψ^	Post-Nargis (Range)^ψ^	
**Annual surveillance data from Myanmar (HMIS)**				

Percentage of general clinic attendance (target 50%) *	15.13 (8.73-26.77)	15.33 (6.26-28.51)	26.15 (13.58-41.92)	21.33 (12.56-36.55)

Percent of child diarrhea cases with severe dehydration	2.37 (0.59-5.45)	1.21 (0.00-4.33)	0.72 (0.00-2.06)	0.92 (0.15-2.43)

Percent child diarrhea cases treated with ORT	97.27 (93.01-100.00)	98.21 (82.40-100.00)	95.69 (59.15-100.00)	97.62 (90.12-100.00)

**Periodic review surveys (Dec 2008, Jul 2009) by Tri-partite Core Group**				

Percentage of population accessing within one hour distance from a health facility			77.00	75.00

Percentage of health facilities with health personnel			91.00	91.00

Percentage of health facilities which have essential drugs for most of the time			76.00	85.00

Ψ Range = township which had lowest percentage/coverage to township which had highest percentage/coverage

HMIS data also show that dysentery was also one of the leading causes of morbidity in the study townships. As shown in Figure 1, dysentery cases peaked sharply in affected areas in 2008 following Cyclone Nargis. Reported incidence of dysentery was 350.9 per 100,000 person-years in 2007; the rate increased to 502.27 in 2008 post-Nargis and fell back to 374.39 in 2009. However, the mortality and case fatality rates for dysentery remained low at <1% reported during 2007-2009.

### Acute respiratory infections (ARI)

Based on data from HMIS, ARI was reported to be the major cause of morbidity and mortality among children under 5 in the ten study townships in Nargis-affected areas. ARI incidence in the Nargis-affected areas typically peaks in June and July (see Figure 1); in 2008, the peak began somewhat earlier--immediately following Cyclone Nargis--and reached higher levels in 2008 compared to 2007 and 2009. Reported ARI incidence in 2007 was 4041.91 per 100,000 persons per year among children under 5, increased greatly to 7279.70 in 2008 following Cyclone Nargis, and dropped back to 4661.59 in 2009. For morbidity among under 5 year-old children, EWAR also reported high numbers of ARI cases during June and August 2008 after Cyclone Nargis incident. Interestingly, ARI mortality was lower in 2008, at 2.76 per 100,000 person-years during the months of 2008 post-Nargis, compared to 4.86 in 2007 and 4.11 in 2009 (Table 2). The case fatality rate for ARI showed a similar declining pattern over the three years: 0.12% in 2007, 0.04% in post-Nargis 2008 and 0.09% in 2009.

### Malaria and dengue

Cases of vector-borne diseases such as malaria and dengue cases decreased significantly in 2009, compared to 2007 and 2008. As shown in Figure 1, there was a significant peak in malaria cases in 2007 but more of a typical seasonal pattern in 2008 following Cyclone Nargis. Reported malaria incidence was 324.44 per 100,000 persons per year in 2007, increased slightly to 372.84 following Cyclone Nargis in 2008, and then dropped to 227.18 in 2009, based on routine HMIS reporting. Similarly, the percentage of malaria patients among general clinic attendance fell from 3.17% in 2007 to 1.63% in 2009. In contrast, the mortality percentage among malaria inpatients (case fatality rate among malaria inpatients) increased, rising from 1.16% in 2007 to 3.31% in 2009. It was shown, however, that malaria morbidity and mortality varied among different townships. From the EWAR report, confirmed malaria cases peaked in July 2008 following Cyclone Nargis, but the monthly pattern of malaria cases shows no significant difference from the seasonal pattern in the study area based on EWAR reporting between June 2008 and May 2009.

**Figure 2:**
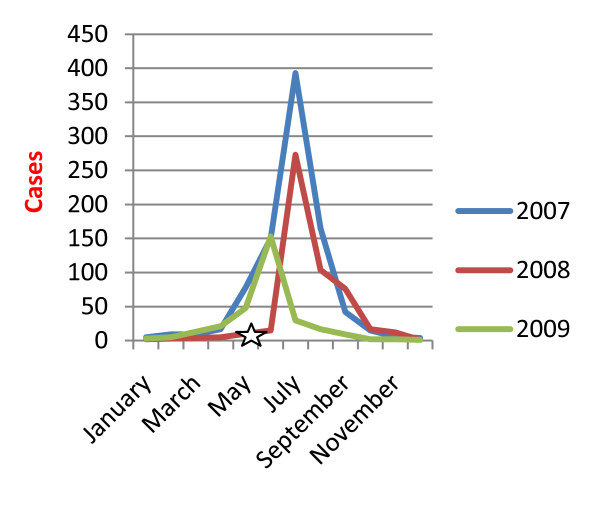
**Reported number of DHF cases**. (White Star): Cyclone Nargis

The incidence rates for reported dengue hemorrhagic fever cases were 35.65 per 100,000 person-years in 2007 and then dropped to 13.03 in pre-Nargis 2008, slightly increased to 15.80 following Cyclone Nargis, and then dropped again to 12.40 in 2009 (Table 2). Dengue hemorrhagic fever cases followed the seasonal patterns during 2007 and 2009 as shown in Figure 2. Confirmed dengue hemorrhagic fever cases reported by EWAR also surged after Nargis, specifically during July 2008, in which there were 273 cases or 0.14% of total consultations. Mortality rate and case fatality rate for dengue hemorrhagic fever decreased between 2007 and 2009, but increased sharply in July 2008 in association with the increase in reported cases.

### Tuberculosis and other diseases

HMIS data also show that morbidity and mortality rates for tuberculosis decreased in the study area between 2007 and 2009 (67.11, 57.23, 57.15, and 47.54 per 100,000 persons per year in 2007, pre-Nargis 2008, post-Nargis 2008 and 2009, respectively; see Table 2). For sexually transmitted infections, incidence rates for genital ulcer (>2 per 100,000 person year) and male urethral discharge (>1 per 100,000 person year) comparable for 2007-2009; thus the disease burden for these STIs was the same before and after the Cyclone Nargis and there were also not much monthly fluctuation cases during 2007-2009. According to EWAR, STIs constituted less than 0.5% of total consultations from June 2008 to May 2009.

Interestingly, as part of the surveillance, we noted that mortality rates for snake bite did not change much during the period from 2007 to 2009, yet case fatality rates for snake bite was quite high in the study area after Cyclone Nargis incident (25.64 in 2008 pre-Nargis vs 36.18 in 2008 post-Nargis). It was also found that vaccine-preventable diseases such as diphtheria, pertussis, neonatal tetanus and measles were not the major causes of morbidity or mortality among the under-5 children during 2007-2009. Incidence rates for viral hepatitis and typhoid were <10 per 100,000 person-years during 2007 and 2009.

### Health services utilization

**Figure 3:**
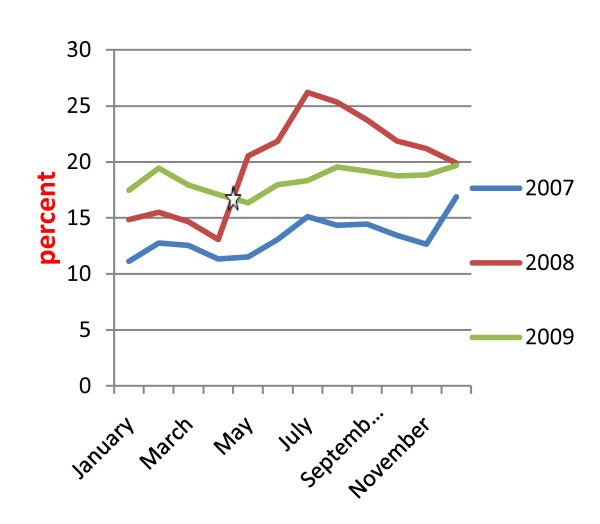
**Monthly general clinic attendance**. (White Star): Cyclone Nargis

Based on the data in HMIS, general clinic attendance (total outpatient consultations in the ten study townships divided by the total population of those townships, expressed as a percentage) was 15.13, 26.15 and 21.33% in 2007, post-Nargis 2008 and 2009, respectively. These figures indicate that general clinic attendance increased significantly in post-Nargis 2008 compared to 2007 (see Table 3). General clinic attendance is an HMIS indicator for health services utilization, The data from EWAR, which includes statistics from international NGOs, indicated that there were a total of 754,852 consultations in 15 townships in the Nargis-affected area one year after the Nargis incident, from June 2008 to May 2009. (Comparable data for the year preceding Nargis are not available.) It was also estimated that the average number of consultations per person during the year following the Nargis incident was about 0.3 visits per person per year, which mean that general clinic attendance percent was 30% and not much different from HMIS data of 26.15% in post-Nargis 2008. As shown in Figure 3, monthly general clinic attendance did increase from May 2008 to July 2008, the 3 months following Cyclone Nargis. Subsequently attendance slowly decreased starting from August 2008. During 2009, monthly attendance appeared to be stable without much variation by month over the year analyzed.

In assessing accessibility to health facilities, TCG survey showed that about 75% of the population in Nargis-affected areas lived within one hour distance from health facilities in post-Nargis 2008 and 2009. It was also found that there was no significant change in presence of health personnel at health facilities (91%) in 2008 to 2009. In contrast, drug availability at health facilities for most of that time dramatically increased from 76% in December 2008 to 85% in July 2009. The drug availability figures are drawn from a TCG survey that asked respondents about drug availability when they went to health care facilities; drug availability is defined as the proportion of health care facilities which have essential drugs most of the time. On the other hand, the average number of clinic visits by household head was 1.9 in July 2009; this falls short when comparing to the international standard target of 2.0-4.0 (as set by Sphere).

### Disease prevention and control measures

During the period from May 2008 to April 2009, there were a total of 50,000 pamphlets, 2,945 posters, and 12,000 booklets about diarrheal diseases and childhood infections distributed to the Nargis-affected areas by National Health Programs and other organizations. Interestingly, most of the participants in focus group discussions pointed out that they were not interested in health education and went to health education centers only for receiving relief items.

"Health education was given on dengue, malaria, diarrhea diseases, and other communicable diseases. Most of the villagers did not go because they were busy with activities such as building shelter." (57 year-old farmer)

Rapid diagnostic test and artemesinin combination therapy (ACT) were available in about 70% of rural and sub rural health centers [15]. One health worker mentioned that *"During Cyclone Nargis, a lot of malaria drugs were given to my health center, but malaria is not prevalent in my area. I was afraid the malaria drugs would expire." *The national health programs and other organizations distributed a total of 282,532 insecticide long-lasting nets (ILLN) for malaria prevention from May 2008 to April 2009, covering about 10% of the population in those areas. Availability of information, education and communication (IEC) materials for dengue in health centers was high, with more than two-thirds of the health centers having IEC materials for dengue after Cyclone Nargis [15]. On the other hand, IEC materials for malaria were present in only about 50% of health centers in Nargis-affected areas in 2009 [15]. The vector-borne diseases control program distributed 37,000 pamphlets on vector-borne diseases from May 2008 to April 2009. In one specific area highly endemic area for malaria, Ngapudaw, the training program for malaria case management was carried out for 80 hospital workers. Insecticide residual spray had also been used in highly endemic areas, covering 40,122 persons, or about 15% of the population, in those areas. Mass mosquito larviciding activities for dengue control were carried out in areas with population coverage of 347,231, or slightly more than 10%, in the study area.

Tuberculosis control programs reached the targets of 70% case detection and 85% treatment success rate before the Nargis incident in the ten study townships. However, it still must be noted, case detection in the Nargis-affected area (at 70%) was already slightly lower than the national average of 77%. Cyclone Nargis negatively affected tuberculosis case detection, which fell from 78% in the months of 2008 before Cyclone Nargis to 64% during the months of 2008 following the incident (see Table 4). The percentage of case detection was quite different among townships, with detection rates lower than 50% in some townships.

**Table 4:** Public health indicators of preventive and control measures in 10 townships of Nargis- affected area, 2007 to 2009

Indicators	2007 (Range)^ψ^	2008		2009 (Range)^ψ^
			
		Pre-Nargis (Range)^ψ^	Post-Nargis (Range)^ψ^	
**Annual surveillance data from Myanmar (HMIS)**				

Average of sanitary latrine coverage (Urban population)	78.44 (43.65-106.40)	N/A	61.78 (29.10-95.65)	82.08 (30.4-107.00)

Average of sanitary latrine coverage (Rural population)	66.53 (12.10-93.46)	N/A	48.42 (20.42-89.35)	71.36 (53.70-93.60)

Average of sanitary latrine coverage (Total Population)	67.99 (22.20-93.20)	75.50 (43.16-95.90)	50.47 (23.05-78.20)	72.36 (56.00-94.80)

Treatment success rate for tuberculosis	90.49 (86.78-97.45)	76.77 (54.28-96.88)	88.59 (81.38-99.42)	N/A

Case detection rate for tuberculosis	69.25 (33.96-155.21)	78.37 (25.60-135.35)	64.64 (20.98-111.11)	N/A

DPT 3^rd ^dose	90.53 (73.1-99.0)	91.8 (78.38-94.7)	90.05 (81.58-96.1)	88.01 (76.2-98.8)

Measles immunization	83.53 (61.4-95.4)	85.51 (68.90-94.77)	89.24 (73.31-95.57)	82.19 (70.8-90.9)

**Periodic review surveys (Dec 2008, Jul 2009) by Tri-partite Core Group**				

Percentage of improved drinking water			N/A	66.00

Percentage of improved sanitation facilities			40.00	43.00

DPT 3^rd ^dose			N/A	66.00

Measles immunization			91.00	88.00

Ψ Range = township which had lowest percentage/coverage to township which had highest percentage/coverage

Coverage of prevention of mother-to-child transmission of HIV (PMTCT) services increased from 2 of the ten study townships in 2007 to 7 such townships in 2009. Performance of PMTCT services improved between 2007 and 2009, for example the percentage of treatment taken by HIV (+) pregnant mothers (61% in 2007 to 80% in 2009) and the resulting percentage of newborns who were HIV (+) (14% in 2007 to <5% in 2009). Despite these improvements, less than 50% of health centers in Nargis-affected areas were found to have HIV IEC materials in 2009 [15]. However, the majority of the surveyed organizations distributed condoms for prevention of STIs; the total number of male condoms distributed by National Health Programs and other organizations was 430,390 following Cyclone Nargis. However, this was still less than the target of 1 condom/person/month recommended by Sphere.

In terms of the Expanded Program on Immunization (EPI), vaccination coverage reached highest levels in 2008 after Cyclone Nargis (see Table 4); coverage for the DPT 3^rd ^dose reached the immunization program target of around 90%, as documented both by routine reporting and TCG survey. However, measles coverage remained lower than the 90% target, with the percentage of 12-23 month-old children inoculated reported at around 84% in 2007 and 2009, but higher during 2008 (86% in the months before Nargis and 89% in the months post-Nargis). The EPI IEC materials were present in around two-thirds of health facilities in Nargis-affected areas as indicated in a survey done by UNICEF during December 2008 [15]. One health professional also mentioned in a focus group discussion that immunization services were successful after Cyclone Nargis.

The sanitary latrine coverage was increased from 78% in 2007 to 82% in 2009 in urban areas and from 67% to 71% in rural areas (see Table 4), however, coverage declined to 62% in urban areas and to 48% in rural areas in the months of 2008 following the Nargis incident. In urban and rural areas combined, the sanitary latrine coverage for the Nargis-affected population during 2008 was 76% in the months pre-Nargis and fell to 51% in the months post-Nargis. However, the sanitary latrine coverage in 2009 was still low in certain rural areas, e.g., in Ngapudaw, Labutta and Bogale townships. The TCG survey reported that the population receiving improved drinking water was 66% in December 2008. That same survey indicated approximately 43% for improved sanitation facilities, which was significantly lower than the MMOH data of 70% in 2009. The large difference in sanitary latrine coverage between the two data sources may be because HMIS is based on population coverage and TCG survey is based on household coverage. Participants in focus group discussions mentioned that sanitary latrine construction by villagers was associated with the supply of both latrine pan and construction cost. *"Latrine pans were distributed. However, I could not construct latrine because I had no money." (25 year-old man) *and *"In our village, most of the villagers were able to construct sanitary latrine because the organization (-----) provided not only gave latrine pans but also provided the construction cost." (18 year-old student)*

## Discussion

MMOH established HMIS as a routine reporting disease surveillance system several years ago, and the system has been fully functioning in almost all levels of hospitals. However, hospitals, especially at the secondary referral level, should consider strengthening microbiological surveillance because of weakness in laboratory capacity at such hospitals. Microbiological surveillance is important for early detection of public health emergencies, especially with regard to communicable disease outbreaks. Currently, there are only about 20-30% of hospitals that have microbiological surveillance systems important for investigation of public health emergencies, compared to 64.5% of hospitals in China [14].

The World Health Organization recommends that emergency surveillance should include bloody diarrhea, acute watery diarrhea and suspected cholera, acute respiratory tract infection (ARI), measles, meningitis, HIV/AIDS, sexually transmitted infections, tuberculosis, and neonatal tetanus [6]. Setting up of EWAR with participation of national and international organizations working in public health emergency response is a best practice for public health emergency management and should be set up as soon as possible as part of public health emergency management. These diseases are comparable to those included in Myanmar's routine surveillance system, but there is room for improvement in areas such as recording and calculation of timeliness for surveillance and sensitivity for outbreak detection [13]. HMIS reporting is a passive surveillance system, which includes such limitations such as under-reporting; potential unreliability because of dependence on basic health staff for data collection, and incompleteness of data due to underutilization and difficulties of accessing health services in some rural areas. However, it is also necessary to set up the database for logistic capacity and drug supply within the health sector in cooperation with other organizations working in public health emergencies [16].

The national surveillance data taken together with the EWAR reports suggested that morbidity of diarrhea, dysentery and ARI increased significantly for about 3 months after Cyclone Nargis, but the incidence of other diseases did not deviate much from normal levels or seasonal patterns, compared to 2007 and 2009. The increases observed during the months after Cyclone Nargis included some, but not all, of the outbreak-prone diseases that have been documented to increase following other disaster incidents elsewhere----ARI, diarrheal diseases, measles, malaria in endemic areas, epidemic meningococcal disease, dengue, tuberculosis, tetanus, pneumonia, relapsing fever, yellow fever, and typhus [6,17]. Early diagnosis and prompt treatment by trained staff that use standard protocols at all health facilities improves the management of communicable diseases and mitigates the health impact of a natural disaster [5]. Thus, the relatively high availability of drugs for common diseases in risk areas and the strengthened preparation for disaster management and health services offered by governmental programs, international organizations and NGOs might help explain why the morbidity and mortality of common communicable diseases were lower than might have been expected after the Nargis incident.

The country still needs to reach its targets for routine disease control programs. For example, the tuberculosis program reached its overall targets of case detection and treatment success rates, but these levels varied in different townships. More efforts are needed in townships that did not reach the national targets. While PMTCT coverage improved during the study period, healthcare providers noted that PMTCT services should be secured at all townships by national health programs with financial and technical support. This involves many stakeholders that manage PMTCT services in the country. While measles vaccination coverage was slightly less than 90% in affected areas and disease incidence in those areas did not increase post-Nargis, it has been suggested that if measles vaccination coverage rates are lower than 90%, measles vaccination should be given priority to prevent an outbreak of measles in emergency situations [5,6]. In all, it was found that coverage for all immunizations was slightly lower after the Nargis incident in 2009 compared to 2008. Immunization services should be restored and sustained as part of the routine National Health Program with involvement of donor agencies and township health departments.

Community awareness programs should be strengthened because community awareness of early treatment and proper case management is essential to reducing the impact of communicable diseases such as diarrheal diseases, ARI, malaria and dengue [6]. Almost all of the organizations surveyed for this study distributed several IEC materials regarding communicable diseases; however, most of the participants in the focus group discussions reported not being interested in health education programs. Evaluation of the effectiveness of these health education programs should be carried out to identify ways to improve such efforts in future emergencies.

Utilization of health services, marked by indicators such as general clinic attendance, improved between 2007 and 2009. However, rates were still quite low in comparison with HMIS target achievement of 50%. Clinic attendance rates of some townships were less than 15% while TCG survey also reported that health services utilization did not reach the Sphere target of 4 visits per person per year [10,11]. It remains necessary to research factors influencing the utilization of health services. Sanitary latrine coverage in Nargis-affected townships was slightly lower than the national sanitary latrine coverage of around 80% and fell sharply following the incident. The distribution of water and sanitation services was quite varied across the affected communities. The water and sanitation program should be strengthened in townships which are below the national average, through cooperation among government and non-government stakeholders and the respective communities.

## Limitations

There are several limitations to this study. First, validity and reliability of secondary data (information bias) may be limited, but these limitations were outside the purview of the research team due to the secondary nature of the data. Population movement in Nargis-affected areas may have resulted in unreliable denominators that were used in the calculation of epidemiological measurements such as incidence, mortality, and service utilization rates. Information bias may be present in the self-administered questionnaires completed by hospital officials. Participants in focus group discussions may not be representative of their respective communities (selection bias). Recall bias may be another problem from the focus groups because the study was carried out 2 years after Cyclone Nargis.

## Conclusion

Compared to what might have been expected, the health impact in Nargis-affected areas was relatively modest. Specifically, incidence rates increased following the incident for diarrhea, dysentery and ARI but not for other diseases, and mortality was largely unchanged for all diseases. Nonetheless, communicable diseases still pose a high burden in these townships and all stakeholders should strengthen the health services to improve service coverage and quality and health outcomes. Water and sanitation services in the study area were already well below the national coverage and were adversely impacted by Cyclone Nargis--these probably contributed to the increases observed of diarrhea and dysentery cases in the months immediately after the incident. Both water and sanitary latrine coverage still need to be improved in Nargis-affected areas to achieve national Millennium Development Goals. Measles immunization was improved immediately after Cyclone Nargis due to the efforts from stakeholders and mass immunization program in affected areas. These efforts may have helped to prevent outbreaks of measles following the incident. However, sustainability of the immunization program is still a challenge in the study area. The establishment of the EWAR surveillance system immediately after the incident by participation of all organizations working in public health emergency response is a best practice for public health emergency management and it is recommended as part of routine disaster management. While health services utilization in the cyclone-affected study area improved after the incident, more effort will be needed for townships with low utilization rates to determine the factors contributing to low utilization such as supply side (e.g., accessibility of the health services) or demand side (e.g., patient factors such as financial difficulties). Especially in light of the disinterest in health education voiced by most participants in the focus group discussions, the impact of risk communication for PHE in Myanmar should also be studied, to determine the effectiveness of IEC materials and activities in the community and identify ways to improve their effectiveness. Lessons learned in terms of strengths and weaknesses for communicable disease prevention control in response to Cyclone Nargis could be applied to policy development, planning and preparedness for management of future public health emergencies in Myanmar.

## Competing interests

The authors declare that they have no competing interests.

## Authors' contributions

NWM, JK, PS were involved in the conceptualization and design of the study. NWM prepared research instruments and other study logistics, and collected data in Myanmar. SLN and TTM assisted in study management and data support in Myanmar. KC, PS, AKM, PP provided conceptual framework and technical support for the study. NWM and JK performed analyses and drafted the manuscript. All authors read and approved the final manuscript.
